# Supplementary material for: Preferences and uptake of home-based HIV self-testing for maternal retesting in Kenya
Source: medRxiv. 2024 Mar 30:2024.03.28.24305050. Preprint. [Version 1] doi: 10.1101/2024.03.28.24305050 (PMC10996825; doi:10.1101/2024.03.28.24305050)

medRxiv preprint doi: https://doi.org/10.1101/2024.03.28.24305050; this version posted March 30, 2024. The copyright holder for this preprint (which was not certified by peer review) is the author/funder, who has granted medRxiv a license to display the preprint in perpetuity.
It is made available under a CC-BY 4.0 International license .

Supplementary Tables and Figures

Table A. Correlates of selecting HB-HIVST as a retesting strategy at enrollment (vs. CB-RDT) (N=994)

	n (%) or median (IQR)				Prevalence Ratio (95% CI)			
	HB-HIVST[b](N=330)		CB-RDT[c] (N=664)		Crude	p	Adjusted	p
Western Kenya	330	168 (51)	664	366 (55)	0.89 (0.72-1.10)	0.29	1.03 (0.70-1.52)	0.89
Age (year)	330	23 (21-27)	664	24 (21-27)	0.99 (0.97-1.01)	0.39	0.97 (0.94-1.00)	0.08
Gestational age ≥24 weeks at enrollment	330	233 (71)	664	491 (74)	0.99 (0.97-1.01)	0.39		
Completed secondary education	330	185 (56)	664	325 (49)	1.21 (1.01-1.45)	0.04*	1.38 (1.04-1.85)	0.03*
Employed	330	104 (32)	664	231 (35)	0.91 (0.75-1.10)	0.31		
Household income ≥10,000 (KSH) per month	295	125 (42)	581	193 (33)	1.29 (1.06-1.58)	0.01*	1.43 (1.02-2.01)	0.04*
Depression[a]	330	166 (50)	664	381 (57)	0.83 (0.69-1.00)	0.05*	0.79 (0.56-1.12)	0.18
Have live births	330	172 (52)	664	375 (56)	0.89 (0.75-1.06)	0.19		
Current pregnancy intended	328	196 (60)	663	386 (58)	1.04 (0.85-1.27)	0.68		
Relationship	330		664					
Married[b]		284 (86)		548 (83)	ref	ref		
No partner		21 (6)		46 (7)	0.92 (0.64-1.32)	0.65		
Not married w partner		25 (8)		70 (11)	0.77 (0.54-1.09)	0.14		
Low partnership power[c]	297	79 (27)	598	147 (25)	1.07 (0.87-1.33)	0.52		
Ever tested with STI	326	5 (2)	662	22 (3)	0.55 (0.25-1.24)	0.15		
Polygamous[+]	309	8 (3)	616	46 (7)	0.43 (0.22-0.82)	0.01*		
Traveling time to clinic ≥1 hour[i]	330	83 (25)	663	125 (19)	1.27 (1.03-1.57)	0.03*	1.39 (0.94-2.06)	0.1
Using transportation to clinic[d]	329	223 (68)	663	473 (71)	0.89 (0.74-1.09)	0.26		
Waiting time ≥1 hour at clinic[d]	330	128 (39)	663	206 (31)	1.25 (1.05-1.49)	0.01*	1.27 (0.91-1.79)	0.16
Ever left clinic because of long wait	329	46 (14)	664	79 (12)	1.13 (0.88-1.44)	0.33		
Schedule not working with clinic hours	330	56 (17)	664	67 (10)	1.45 (1.16-1.81)	<0.01*	2.26 (1.44-3.57)	<0.01*

Interquartile range (IQR); home-based self-testing (HB-HIVST); clinic-based testing (CB-RDT); prevalence ratio (PR); confidence interval (CI). a. score of >10 on Edinburgh Postnatal Depression Scale (EDPS); b. married / cohabitating (vs. no partner); c. score in lowest quantile (<2.15) on Sexual Relationship Power Scale (SRPS); d. at last clinic visit before enrollment
* p<0.05; ** Includes as interaction term with preterm birth. Kenya Shilling (KSH) ~ $1 USD.
+. Polygamy was excluded in the adjusted model due to missing data

medRxiv preprint doi: https://doi.org/10.1101/2024.03.28.24305050; this version posted March 30, 2024. The copyright holder for this preprint (which was not certified by peer review) is the author/funder, who has granted medRxiv a license to display the preprint in perpetuity.
It is made available under a CC-BY 4.0 International license .

Table B. Correlates of completing HIV retesting with HB-HIVST (vs. CB-RDT) among women who selected HB-HIVST at enrollment (N=295)[#]

	Retested with HB-HIVST (N=121)		Retested with CB-RDT (N=174)		Crude PR (95% CI)	p
	N, median (IQR) or n (%)					
Western Kenya	121	47 (39)	174	91 (52)	0.71 (0.53-0.95)	0.02*
Age (years)	121	24 (22-27)	174	23 (21-27)	1.01 (0.99-1.04)	0.31
Gestational age ≥24 weeks at enrollment	121	86 (72)	174	129 (74)	0.94 (0.69-1.28)	0.70
Preterm birth (<37 weeks gestation age at delivery)	120	27 (22)	174	70 (40)		
Tested in pregnancy					0.83 (0.35-1.94)	0.67
Tested in postpartum					0.29 (0.15-0.57)	<0.001*
Tested during postpartum (ref: pregnancy/delivery)	120	84 (70)	174	99 (57)	**	**
Completed secondary education	121	67 (55)	174	102 (59)	0.91 (0.69-1.20)	0.51
Employed	121	41 (34)	174	56 (32)	1.06 (0.79-1.42)	0.70
Household income ≥10,000 (KSH) per month	107	56 (52)	158	62 (39)	1.40 (1.04-1.87)	0.03*
Depression[a]	121	47 (39)	174	97 (56)	0.64 (0.48-0.86)	<0.001*
Have live births	121	73 (60)	174	88 (51)	1.25 (0.94-1.66)	0.13
Current pregnancy intended	121	74 (61)	172	103 (60)	1.02 (0.78-1.32)	0.89
Married/cohabitating[b]	121	109 (91)	174	148 (85)	1.47 (0.88-2.44)	0.14
Relationship duration <1 year[b]	116	10 (9)	162	22 (14)	0.73 (0.44-1.21)	0.22
Low partnership power[c]	117	31 (26)	161	43 (27)	1.02 (0.74-1.40)	0.92
Traveling time to clinic ≥1 hour[d]	121	27 (22)	174	49 (28)	0.84 (0.6-1.16)	0.29
Using transportation to clinic[d]	120	75 (62)	174	122 (70)	0.81 (0.61-1.07)	0.14
Waiting time ≥1 hour at clinic[d]	121	51 (42)	174	64 (37)	1.16 (0.88-1.53)	0.30
Ever left clinic because of long wait	121	21 (17)	174	20 (12)	1.31 (0.92-1.87)	0.14
Schedule not working with clinic hours	121	19 (16)	174	27 (16)	1.02 (0.69-1.49)	0.93
Partner tested for HIV during follow-up[e]	115	84 (73)	155	82 (53)	1.70 (1.22-2.36)	<0.001*

Home-based self-testing (HB-HIVST); clinic-based testing (CB-RDT); Interquartile range (IQR); prevalence ratio (PR); confidence interval (CI); a. assessed by Edinburgh Postnatal Depression Scale (EDPS) with a score of >10; b. married / cohabitating (vs. no partner); c. score in lowest tertile (<2.15) on Sexual Relationship Power Scale (SRPS); d. assessed with the last clinic visit before enrollment; e. among women who had an HIV-negative or unknown partner and reported partner testing status during follow-up. Kenya Shilling (KSH) ~ $1 USD.
* p<0.05; ** Includes as interaction term with preterm birth

medRxiv preprint doi: https://doi.org/10.1101/2024.03.28.24305050; this version posted March 30, 2024. The copyright holder for this preprint (which was not certified by peer review) is the author/funder, who has granted medRxiv a license to display the preprint in perpetuity.
It is made available under a CC-BY 4.0 International license .

Table C. Correlates of completing HIV retesting with HB-HIVST (vs. CB-RDT) by 14 weeks postpartum among women who completed retesting with a follow-up visit within 3 months of the estimated 14 weeks postpartum date (N=813)

	Crude PR (95% CI)	p-value	Adjusted PR (95% CI)	p-value
Western Kenya site (vs Nairobi site)	0.70 (0.49-0.99)	0.04*	1.13 (0.64-2.02)	0.67
Age (years)	1.02 (0.99-1.06)	0.15	1.02 (0.97-1.07)	0.51
Gestational age ≥24 weeks at enrollment	0.90 (0.64-1.27)	0.56		
Secondary education completed	1.12 (0.80-1.57)	0.50		
Employed	1.21 (0.86-1.69)	0.27		
Household income ≥10,000 (KSH) per month	1.72 (1.21-2.44)	<0.01*	1.57 (0.99-2.49)	0.05
Tested during postpartum (ref: pregnancy)	1.16 (0.81-1.66)	0.41	**	**
Preterm birth (<37 weeks gestation age at delivery)	0.58 (0.38-0.87)	0.01*		
Tested in pregnancy			0.96 (0.37-2.49)	0.94
Tested in postpartum			0.32 (0.17-0.60)	<0.001*
Depression[a]	0.51 (0.36-0.72)	<0.01*	0.40 (0.24-0.67)	<0.001*
Have live births	1.09 (0.78-1.52)	0.61		
Current pregnancy intended	0.99 (0.70-1.38)	0.93		
Married/cohabitating[b]	1.68 (0.96-2.95)	0.07	1.53 (0.58-4.03)	0.39
Relationship duration <1 year	0.63 (0.33-1.21)	0.17		
Low partnership power[c]	1.06 (0.72-1.56)	0.77		
Ever diagnosed with STI	0.36 (0.05-2.42)	0.29		
Traveling time to clinic ≥1 hour[d]	0.83 (0.54-1.28)	0.40		
Used transportation to travel to clinic[d]	0.75 (0.53-1.06)	0.11		
Waiting time ≥1 at clinic[d]	1.37 (0.99-1.92)	0.06	1.31 (0.84-2.05)	0.23
Ever left clinic because of long wait	1.48 (0.97-2.25)	0.07	1.56 (0.84-2.87)	0.16
Schedule not working with clinic hours	1.45 (0.92-2.28)	0.11		
Partner tested for HIV during follow-up[e]	2.96 (2.03-4.31)	<0.01*	4.67 (2.71-8.04)	<0.001*

Prevalence ratio (PR); confidence interval (CI); a. assessed by Edinburgh Postnatal Depression Scale (EDPS) with a score of >10; b. married / cohabitating (vs. no partner); c. score in lowest quantile (<2.15) on Sexual Relationship Power Scale (SRPS); d. assessed with the last clinic visit before enrollment; e. among women who had an HIV-negative or unknown partner and reported partner testing status during follow-up. Kenya Shilling (KSH) ~ $1 USD.
* p<0.05; ** Included as interaction term with preterm birth

medRxiv preprint doi: https://doi.org/10.1101/2024.03.28.24305050; this version posted March 30, 2024. The copyright holder for this preprint (which was not certified by peer review) is the author/funder, who has granted medRxiv a license to display the preprint in perpetuity.
It is made available under a CC-BY 4.0 International license .

Figure A. Future test preference among women who completed follow-up within 3 months of the estimated 14-week postpartum date, by retesting type at follow-up (N=813). Questions were answered based on self-reported responses of “Strongly Agree” or “Agree”. * $p<0.05$ by Chi-square test. Home-based HIV self-test (HB-HIVST); clinic-based rapid diagnostic test (CB-RDT).

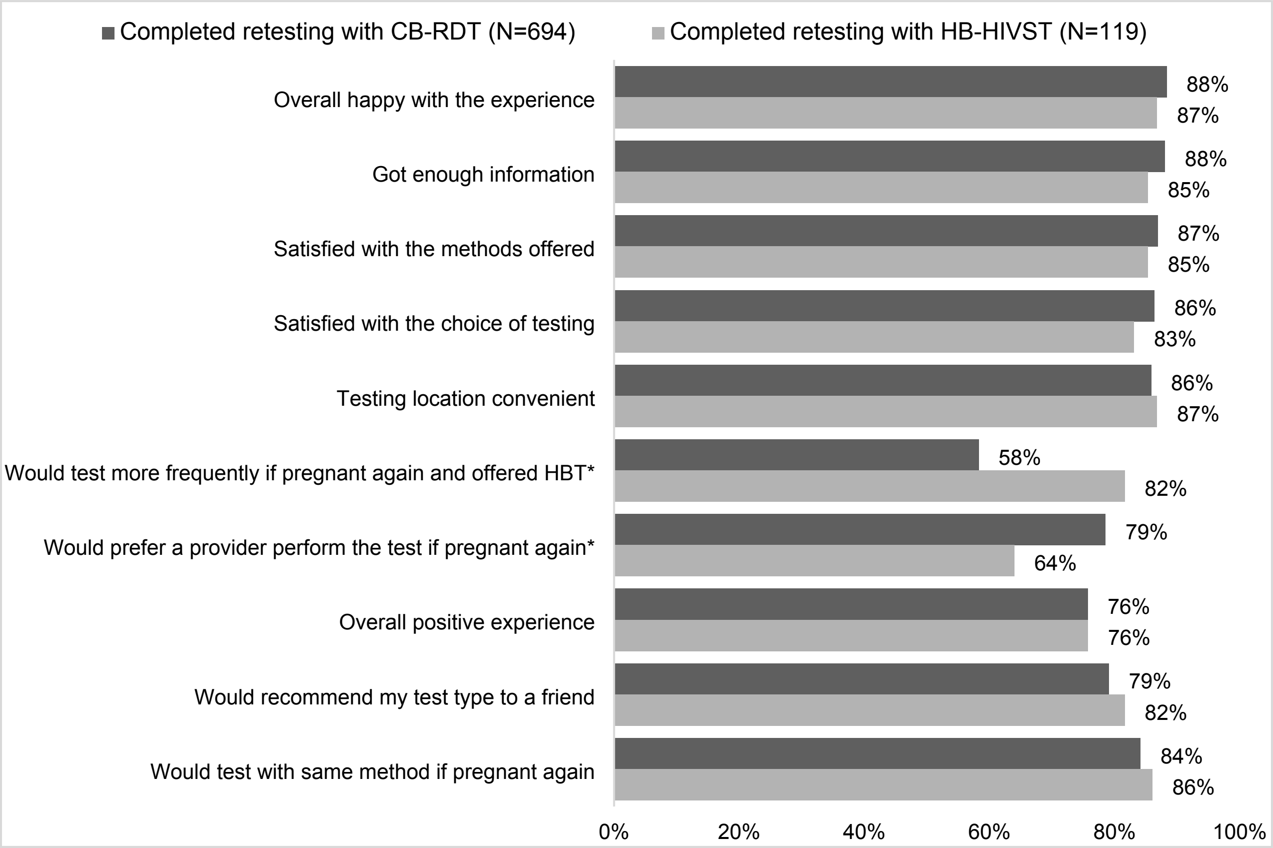

Supplement: Supplement 1 [file NIHPP2024.03.28.24305050v1-supplement-1.pdf]
